# A fast and accurate brain extraction method for CT head images

**DOI:** 10.1186/s12880-023-01097-0

**Published:** 2023-09-12

**Authors:** Dingyuan Hu, Hongbin Liang, Shiya Qu, Chunyu Han, Yuhang Jiang

**Affiliations:** School of Mechanical Engineering and Automation, University of Science and Technology Liaoning, NO.185 in Qianshan Middle Street, Anshan, 114000 Liaoning Province PR China

**Keywords:** Fully convolutional neural network, Threshold segmentation, Head CT images, Brain extraction

## Abstract

**Background:**

Brain extraction is an essential prerequisite for the automated diagnosis of intracranial lesions and determines, to a certain extent, the accuracy of subsequent lesion recognition, location, and segmentation. Segmentation using a fully convolutional neural network (FCN) yields high accuracy but a relatively slow extraction speed.

**Methods:**

This paper proposes an integrated algorithm, FABEM, to address the above issues. This method first uses threshold segmentation, closed operation, convolutional neural network (CNN), and image filling to generate a specific mask. Then, it detects the number of connected regions of the mask. If the number of connected regions equals 1, the extraction is done by directly multiplying with the original image. Otherwise, the mask was further segmented using the region growth method for original images with single-region brain distribution. Conversely, for images with multi-region brain distribution, Deeplabv3 + is used to adjust the mask. Finally, the mask is multiplied with the original image to complete the extraction.

**Results:**

The algorithm and 5 FCN models were tested on 24 datasets containing different lesions, and the algorithm’s performance showed MPA = 0.9968, MIoU = 0.9936, and MBF = 0.9963, comparable to the Deeplabv3+. Still, its extraction speed is much faster than the Deeplabv3+. It can complete the brain extraction of a head CT image in about 0.43 s, about 3.8 times that of the Deeplabv3+.

**Conclusion:**

Thus, this method can achieve accurate brain extraction from head CT images faster, creating a good basis for subsequent brain volume measurement and feature extraction of intracranial lesions.

**Supplementary Information:**

The online version contains supplementary material available at 10.1186/s12880-023-01097-0.

## Background

Computed tomography (CT) is widely used to detect intracranial lesions in humans. Domestic and international researchers have been developing automated diagnostic systems for intracranial lesions based on CT to assist radiologists in making accurate and rapid diagnoses [1–4]. However, not all the information in a set of head CT images is useful; for example, diagnostic equipment, pillows, skulls, and other non-brain tissues are not useful for the diagnosis of intracranial lesions and can significantly affect the feature extraction of intracranial lesions by automated diagnostic systems. Extracting the brain from CT images can provide a better environment for subsequent feature extraction of intracranial lesions [5], which determines the performance of subsequent intracranial lesion detection to a certain extent. Therefore, improving the accuracy and speed of extracting the brain is significant. However, the complexity of CT images of the head, such as the unclosed skull, the distribution of multi-region of the brain, and the different characteristics of different lesions, make high-quality brain extraction difficult [6].

In recent decades, researchers at home and abroad have researched brain extraction and proposed representative algorithms, roughly divided into traditional image segmentation methods, secondary development of medical image post-processing software, and deep learning models. Traditional image segmentation methods achieve the segmentation of target regions by artificially set rules. MM Kyaw et al. [7] used a tracking algorithm to perform brain parenchyma extraction, but extracranial soft tissue could not be eliminated. B Shahangian et al. [8] used threshold segmentation, median filtering, and image and mask multiplication for brain extraction. They later built on this to achieve further segmentation of cerebral hematomas with high accuracy. N Farzaneh et al. [9] used a custom distance regularized level set evolution (DRLSE) for brain extraction before further implementing subdural hematoma segmentation. Anjali Gautam et al. [10] and G Cao’s team [11] clustered images using WMFCM (White Matter Fuzzy C-means) and FCM (Fuzzy C-means), respectively, and then used morphological imaging to carry out the extraction of brain parenchyma. Soumi Ray et al. [12] designed a specific automatic seed point selection method for the region growing method and skillfully utilized the propagation of the brain mask to extract the brain, achieving high segmentation speed and decent accuracy. However, there are still some limitations for basis cranii layer images. Combining the above statements while considering the properties of traditional image segmentation methods, it is difficult to simultaneously overcome large extracranial soft tissue edema, unclosed skull, multi-regional distribution of the brain, and complexity of basis cranii layer image structure using traditional image segmentation methods.

The secondary development of medical image post-processing software is widely used in magnetic resonance ( MR ) and CT. Mitchell et al. [13]modified the fractional intensity (FI) parameters. They adjusted the brain parenchyma threshold range based on the Brain Extraction Tool (BET) to achieve high accuracy brain extraction of MR and CT images. Bauer et al. [14]developed a brain extraction method based on Insight Toolkit (ITK), which first forms a rough mask [15, 16] based on the original image and later uses a level set algorithm to increase the accuracy further. This method is already available for public use. However, integrating both ways into other systems will take much work.

Recently, deep learning has also been widely used in brain neuroimage. DHM Nguyen et al. [17] combined the active shape model and convolutional neural network (CNN) to give full play to the advantages of two to extract the brain from head images with good results. Zeynettin Akkus et al. [18] proposed a full convolutional neural network (FCN) based approach and tested five models, including 2D U-Net, two modified versions of 2D U-Net, 3D U-Net, and SegNet. The experimental results show that the best model has strong robustness and high accuracy, which proves the feasibility of FCN to achieve CT image brain extraction. However, the effects of different lesions on the FCN segmentation effect still need to be thoroughly tested. In addition, the segmentation speed of FCN is relatively slow, and further improvements in segmentation speed while ensuring accuracy and robustness have yet to be thoroughly tried.

Here, a fast and accurate brain extraction method, FABEM, is proposed for the problem of slow FCN segmentation in head CT images. And test the algorithm and five FCN models on 24 sets of head CT images containing different lesions. To the best of our knowledge, this is the first test on a large number of head CT images containing various lesions, which better validates the practicality of the algorithm. The experimental results demonstrate that the method’s overall performance is better than previous algorithms. The contributions of this paper are as follows: an integrated algorithm is proposed to give full play to the advantages of traditional methods and FCNs to have faster extraction speed while maintaining good robustness and accuracy in the brain extraction task of head CT images; the performance of five FCN models in the brain extraction task is evaluated; and the effects of multiple lesions on the segmentation effects of the algorithms are explored.

## Materials and methods

### Data selection and processing

The datasets were derived from the RSNA Intracranial Hemorrhage Original Size PNGs (RIHOSP)dataset, publicly available on the Kaggle website, and the CQ500 dataset, publicly available on the Academic Torrents website. No patient privacy was involved.

With the assistance of radiologists, we extracted three sets of images from these two datasets. First, 5017 slices were selected from the RIHOSP dataset as the first set of images, of which 2063 were single-region distribution images of the brain, of which 540 were images of the basis cranii layer, and a total of 1523 were images of other layers, the remaining 2954 were multi-region distribution images. Then 140 sets of head CT images were randomly screened from the RIHOSP dataset as the second set of images, with an average of 25 slices per set, some of which contained intracranial hematomas, soft tissue edema, and other lesions. In the third group, 24 sets of head CT images containing different lesions were screened from the CQ500 dataset, with 16 slices in each group. Among the first 20 groups containing lesions, there were 10 cases of intracranial hematoma, 2 cases of each subtype; 3 cases of cerebral infarction; 2 cases of skull fracture; 2 cases of soft tissue edema; 2 cases of physiologic calcification; and 1 case of the intracranial cyst. The latter four groups had no lesions, 2 in adults and 2 in minors. The CT slice size was 512 × 512 × 3, and each group’s slices that did not contain brain parenchyma were removed. No preprocessing was performed on the remaining head CT images. The experimental operating system is Windows 11, the processor is AMD Ryzen 7 5700 × 8-Core Processor, the graphics card is NVIDIA GeForce 3060Ti, which has 8GB memory for processing data, and the experimental platform is chosen as MATLAB2022b with CUDA version 12.0.

### Fast and accurate brain extraction method

The method skillfully combines the algorithms by using specific detection mechanisms, a cycle structure, and an automatic seed point selection method, achieving good robustness, accuracy, and segmentation speed. The algorithm can be broadly divided into 4 parts: (1) according to the characteristics of the whole set of head CT images, generate stage 1 mask by threshold segmentation, image filling, median filtering, and then multiply it with the original image to generate stage 2 mask; (2) combining closure operations, CNNs, and specific cycle structures to achieve closure of the skull gap ensures complete filling of the stage 1 mask and enhances the robustness of the algorithm; (3) detect the number of connected regions of stage 2 mask, if the number of connected regions is equal to 1, stage 2 mask is directly used as the final mask, and if not, the original image is discriminated; (4) For images whose category is single-region distribution of the brain, the existing stage 2 mask is further segmented using the region growth method [19] to generate the final mask, and for images whose category is multi-region distribution, the FCN model is used to segment and generate a new mask as the final mask, and finally the original image is multiplied with the final mask to complete the extraction of the brain.

#### Preliminary segmentation of brain tissue

**Fig. 1:**
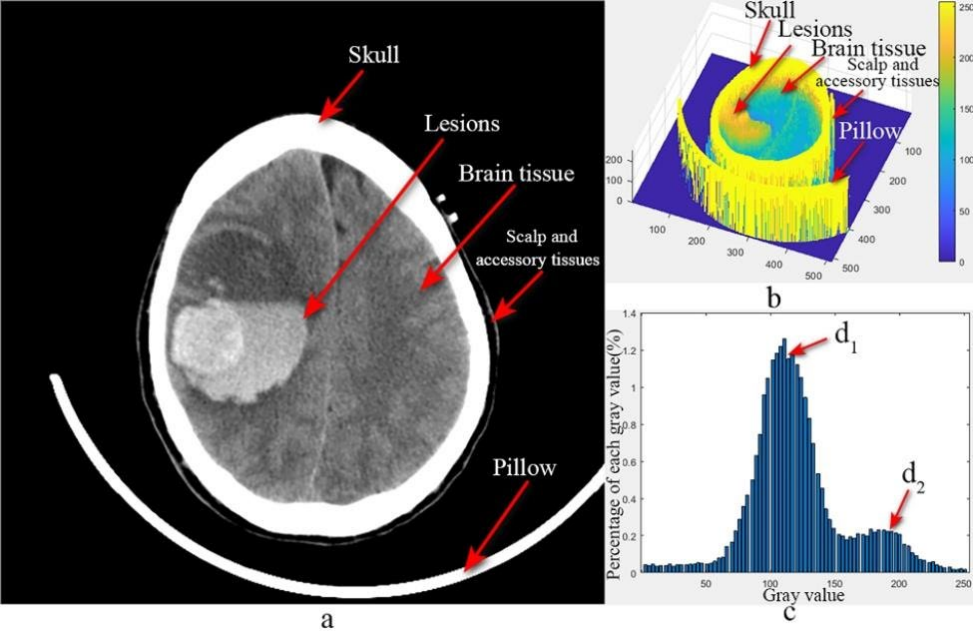
**a**) Original image **b**) Mesh surface of the gray value **c**) Percent bar-chart of gray value from 1 to 254

In CT, images of human tissues are formed based on the absorption properties of radiation energy by human tissue [20, 21]. As shown in Fig. 1(a) shows the original image, and the skull, pillow, scalp, and accessory tissues are the parts to be removed. Figure 1(b) and Fig. 1(c) shows the gray value grid surface plot and the gray value (1-254) percentage bar plot of the original image, respectively. The first peak, d1 in Fig. 1(c), corresponds to the gray value distribution of normal brain parenchyma, and the second peak, d2, corresponds to the gray value distribution of cerebral hematoma. Combining Fig. 1(b) and Fig. 1(c), it can be seen that the gray value of each tissue has a Gaussian distribution, with the skull having the largest gray value at around 255, the intracranial hematoma having the second largest gray value, and the gray value of the brain parenchyma and the extracranial soft tissue are close, both of which are much lower than those of the skull.

Comprehensive analysis of the above, the CT image is first converted into a gray image, and then the skull and brain parenchyma are segmented using threshold segmentation. Considering the influence of CT window width and window level, the threshold range is enlarged to a certain extent. The specific formula is as follows:

1 $${{\text{e}}_1}({\text{i}},j) = \left\{ \begin{gathered}1\,E(i,j)\, \geqslant Max(E) - 15 \hfill \\0\,E(i,j)\, < Max(E) - 15 \hfill \\ \end{gathered} \right.$$

2 $${{\text{e}}_2}(i,j) = \left\{ \begin{gathered}{e_2}(i,j) = E(i,j)\,\,1 \leqslant E(i,j)\, \leqslant Max(E) - 20 \hfill \\{e_2}(i,j) = 0\,E(i,j) > Max(E) - 20\,{\text{or}}\,E(i,j)\, < 1 \hfill \\ \end{gathered} \right.$$

where, *E* represents the gray image;

*max (E)* represents the maximum gray value in the gray image;

*e*_*1*_ represents the skull image after threshold segmentation;

*e*_*2*_ represents the image of skull removal.

**Fig. 2:**
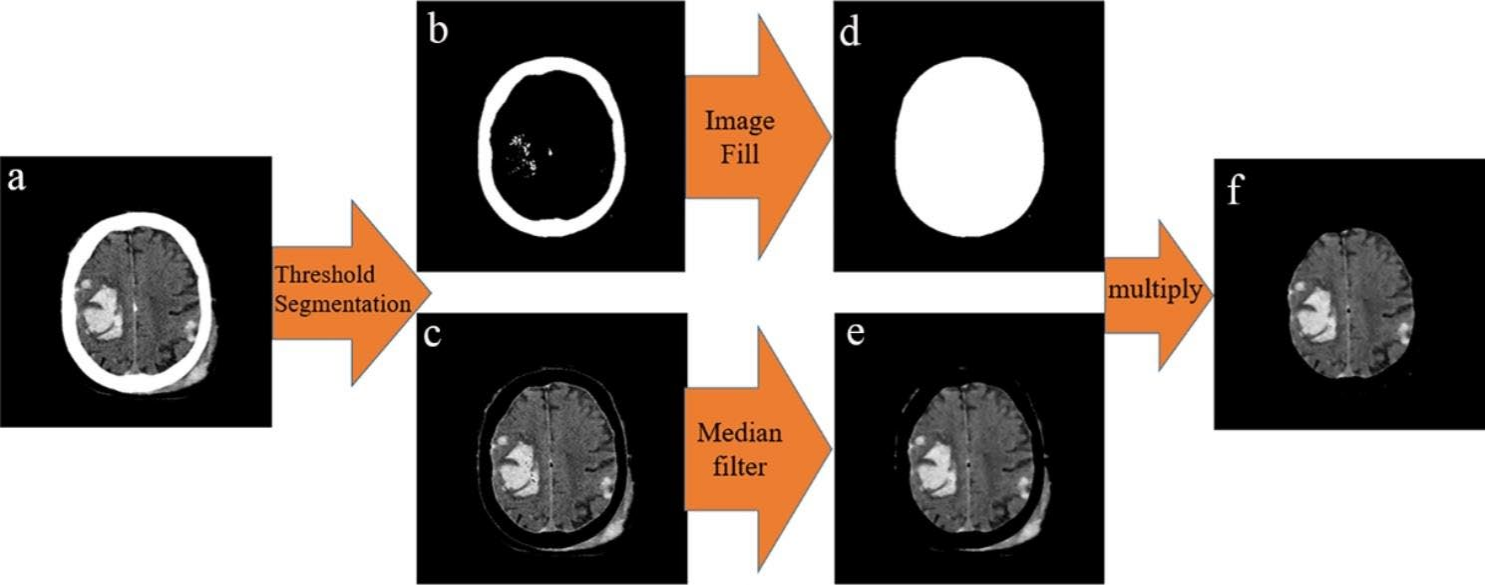
**a**) Original image **b**) Skull image **c**) Image after Removed skull **d**) Image of stage 1 Mask **e**) Denoised image **f**) Preliminary segmented image

The extracted skull is filled as a template to obtain the stage 1 mask, and then the image of the skull removal is multiplied by the stage 1 mask to remove the skull and extracranial soft tissue. The process is shown in Fig. 2.

#### Filling detection and skull closure

**Fig. 3:**
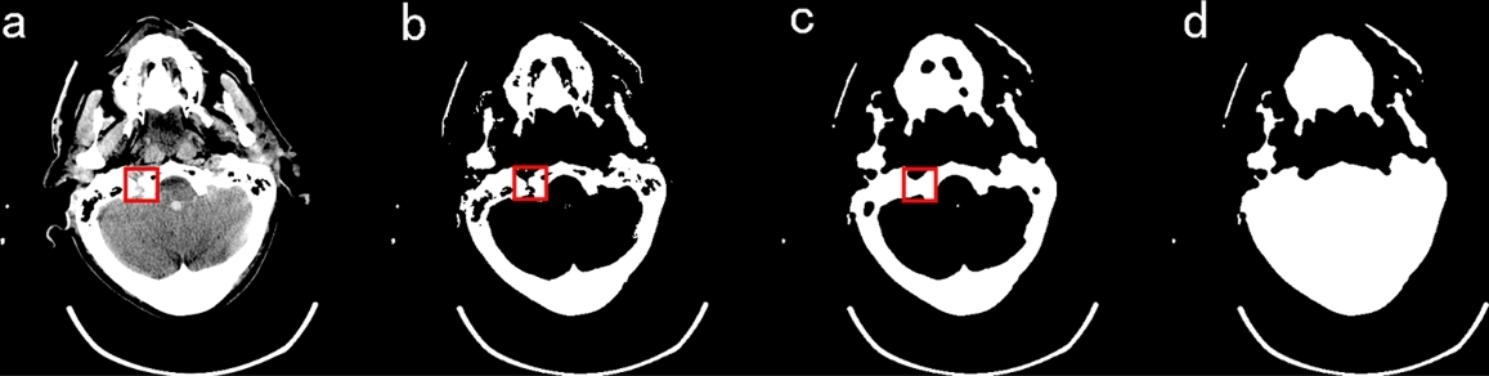
**a**) Original image **b**) Skull image **c**) Images after skull closure **d**) Image of stage 1 Mask

**Fig. 4:**
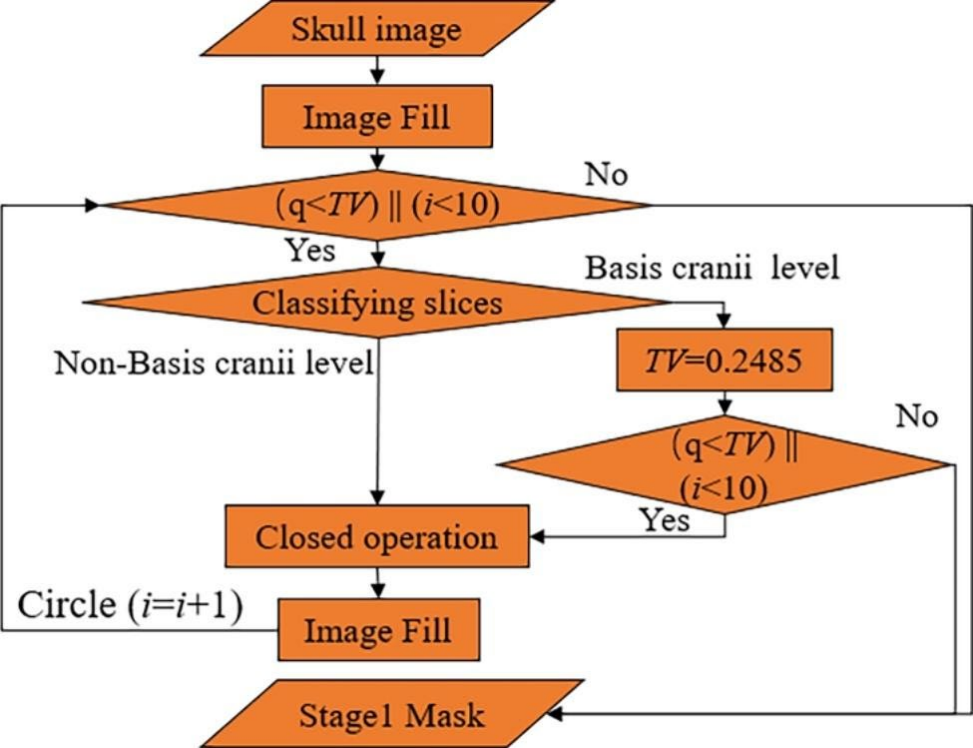
The cycle structure

As seen above, the stage 1 mask is obtained by filling the segmented skull. However, from a tomographic anatomical point of view, not all of the skull is completely closed in a set of head CT images due to the presence of bony seams in the human skull and trauma-induced skull fractures, among other conditions. As shown in Fig. 3(a) and Fig. 3(b), the skull is not closed in the head CT images, and there are obvious gaps in the skull after the threshold segmentation, which cannot guarantee the complete filling of the subsequent stage 1 mask. In this paper, the closure of the skull gap is achieved by the closed operation. Considering that the size, location, and shape of the skull gap are changing, the cycle structure is designed in this paper, which is shown in Fig. 4. Among them,

3 $$q = ({S_i} - {S_{e1}})/{S_i}\,\,\,\,\,\,\left( {0 \leqslant i \leqslant 10} \right)$$

In the formula, *i* represents the number of cycles;

*S*_*i*_ represents the mask area after the *i* th cycle;

*S*_*e1*_ denotes the area of the skull,

*S*_*i*_*-S*_*e1*_ denotes the filled area of the *i* th cycle;

*q* denotes the percentage of the filled area to the mask area after the *i* th cycle.

The extracted skull is first filled once, and whether the mask is filled completely is judged by whether *q* is greater than the threshold value (*TV*), where *TV* is obtained by regression fitting from the soft tissue area and the skull area. Observation and regression experiments were performed on 140 groups of head CT images (3131 sheets). It was found that the proportion of brain tissue area in the soft tissue area at the basis cranii layer was small, and the fitting effect was poor. The average value of brain area as a percentage of the complete mask was 0.2485. The proportion of brain tissue area in the other layers was larger, and the fitting effect was good, with a similarity coefficient of 0.9403 and a p-value of 0 for the statistic, proving that the regression model was established. Therefore, when *q* is less than the *TV* value, then the image is discriminated using CNN. If it belongs to the basis cranii layer, *TV* is reassigned to 0.2485, after which the relationship between q-value and *TV* is judged again, and if it belongs to other layers, closed operation, and refilling are performed directly. The structural element of the closed operation increases one by one during the cycle. If *q* is greater than *TV*, the cycle is jumped out, and the following steps are continued. It is also observed that a small portion of images with a small brain area exists, and even if a complete filling is obtained, *q* is still less than this *TV*. To avoid falling into a dead cycle, the maximum number of cycles is limited at the same time. The closed operation is tested on 140 sets of head CT images. When the number of cycles reaches 8, all the images containing gaps are closed, but the increase in the number of closed operations will bring errors to the subsequent segmentation, and we set the maximum number of cycles to 10 in careful consideration. As shown in Fig. 3(c), the gap of the skull is closed. After filling, the stage 1 mask is obtained, as shown in Fig. 3(d).

We used five CNN models to classify the original images into a total of three classes, where images with the multi-region distribution of the brain are in a separate category, and images with the single-region distribution of the brain are in two types, one for the basis cranii layer and one for the other layers. The first three networks are the AlexNe [22], VGG19 [23], and RestNet502 [24] networks, where the network input layers are not resized, and the image size is converted to the corresponding size of the original network using bilinear interpolation [25] before image input. The fourth is a modified version of the RestNet50 network, with the input layer resized to 512 × 512 × 3 and otherwise unchanged. The fifth one is a modified version of the AlexNet network, which resizes the input layer to 512 × 512 × 3 and uses two batch normalization layers instead of the local normalization layer inherent to the AlexNet network to further speed up model convergence. All networks use the softmax function at the end to generate three types of outputs.

#### Generation of the final mask

**Fig. 5:**
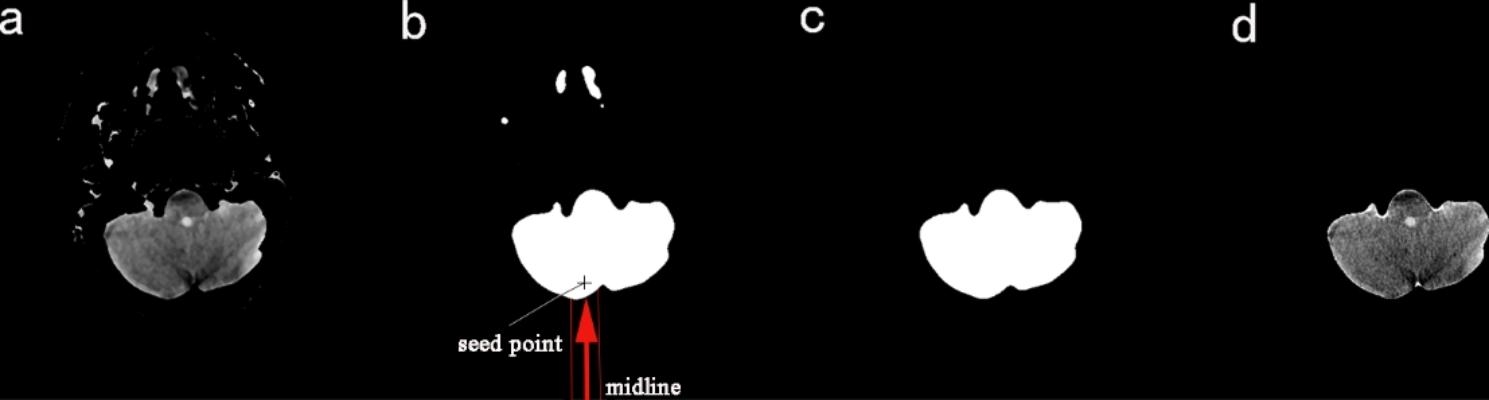
**a**) Initial segmented image **b**) Image of stage 2 mask **c**) Final mask image **d**) Final brain extraction image

In the whole set of head CT sections, the structure of the bottom section is relatively more complex, and it is challenging to ensure high-quality brain extraction by preliminary segmentation only, as shown in Fig. 5(a) through preliminary segmentation, there are still non-brain tissues that are not removed and need further detection and segmentation. We perform median filtering on the initial segmented image to ensure detection accuracy to produce a stage 2 mask, as in Fig. 5(b). Then, the connected component labeling method measures the number of connected regions of the stage 2 mask [26]. If the number of connected regions equals 1, the stage 2 mask is used as the final mask. On the contrary, the stage 2 mask is further adjusted by different methods according to the classification results of the CNN.

For the single-region distribution images of the brain, the stage 2 mask is trimmed using the region growth algorithm. After much observation, In the stage 2 mask, the part to be eliminated originates from the human tissue above the brain parenchyma and not below the brain parenchyma, and the previous steps have eliminated the skull below the brain parenchyma and the extra-cranial soft tissues. Moreover, the brain is distributed in the middle of the image. Then, the first point that is not ‘0’ can be used as the seed point for the region growth algorithm by searching from bottom to top within a certain range of the image midline. To ensure the robustness of segmentation, we moved the position of this point up another five lines to ensure that the seed point can fall precisely in the target region, see Fig. 5(b) for details, and the seed point falls accurately in the region corresponding to the brain parenchyma. After capturing the seed points, the region growing algorithm is used to realize the re-segmentation of the stage 2 mask, and then the final mask is obtained, as in Fig. 5(c). After that, the original image is multiplied with the final mask, and then the brain parenchyma extraction is completed, as in Fig. 5(d). For images with the multi-region distribution of the brain, we use FCN to identify the brain tissue, then perform logical operations on the identification results to generate a new mask, and then use the new mask as the final mask to achieve brain extraction.

We use five FCN models to map the input image to the output mask. The first model uses a 2D U-net [27] structure and the rectified linear unit (ReLU) activation function. The encoding part consists of ten 3 × 3 convolutional layers and four maximum pooling layers, and the decoding part consists of nine 3 × 3 convolutional layers and four deconvolutional upsampling layers, with a softmax function at the end to generate two types of output (brain tissue and non-brain tissue). The second model uses the Segnet [28] structure and the ReLU activation function. The encoding part contains ten 3 × 3 convolutional and batch normalization layers and five maximum pooling layers, and the decoding part has ten 3 × 3 convolutional and batch normalization layers and five upsampling layers. The latter three models all use the Deeplabv3+ [29] structure, and the encoding part comprises a backbone extraction network and an atrous spatial pyramid pooling. In the decoding part, the feature map obtained from the encoding part is upsampled four times, then spliced with the feature map obtained from the backbone extraction network, and finally undergoes one 3 × 3 convolution and four times upsampling. The latter three models differ in the backbone extraction networks, which are ResNet50, Mobilenetv2, and Xception for the fourth model to the fifth model, respectively.

**Fig. 6:**
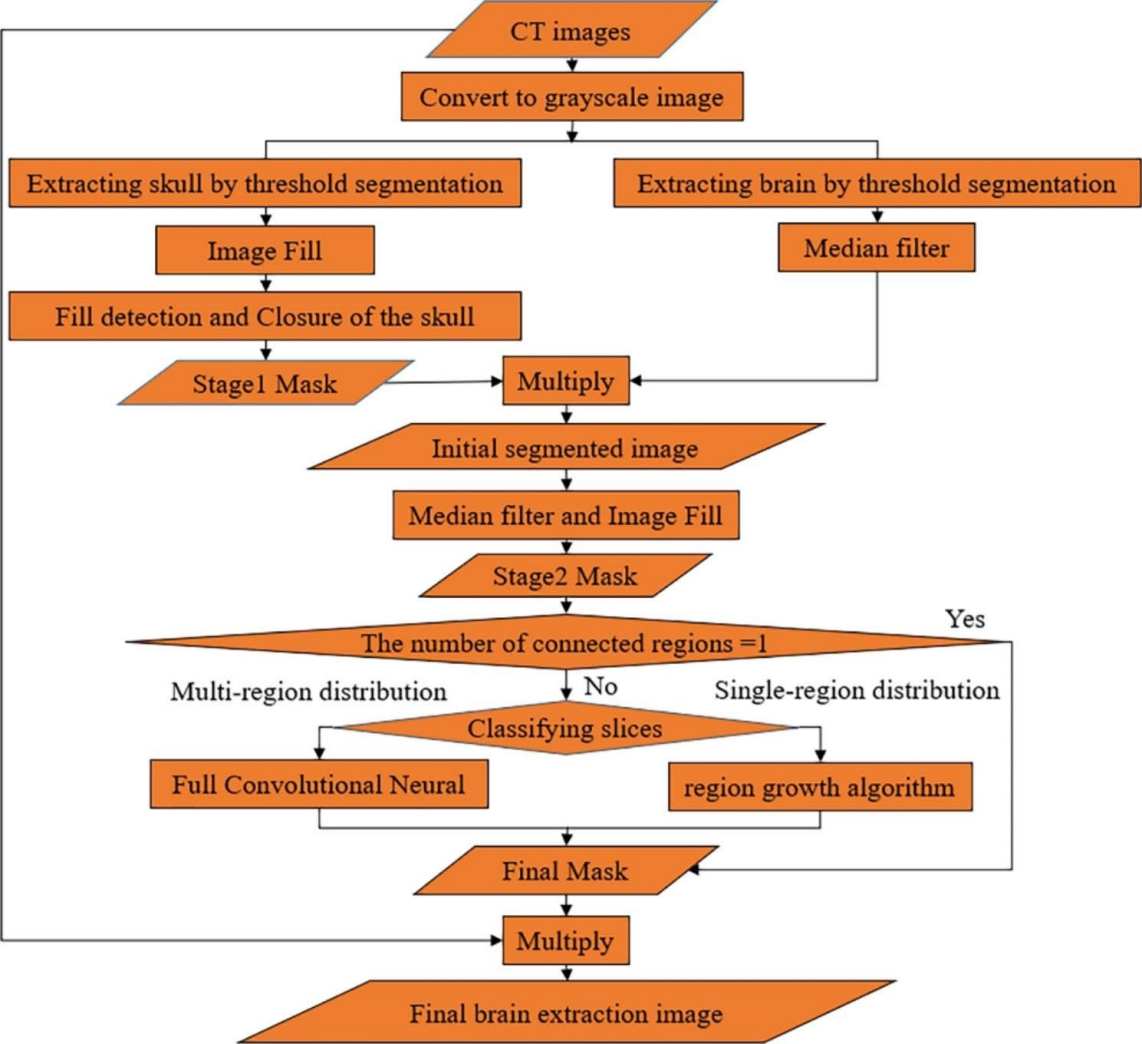
Flowchart of FABEM

In summary, the overall flowchart of the FABEM is shown in Fig. 6. The soft tissue and skull are separated by threshold segmentation first, and then the image is filled with the skull as the template to get the stage 1 mask. The fill detection and closed operations are designed to overcome the problem of skull gaps, further ensure the fill integrity of the stage 1 mask, and increase the algorithm’s robustness. The multiplication of segmented soft tissue and stage 1 mask is then used to complete the initial segmentation. Median filtering eliminates small areas of non-brain tissue in the image throughout the algorithm. The connected component labeling method determines whether further adjustment of the stage 2 mask is required, and the region growing algorithm and FCN are used for further adjustment of the stage 2 mask. In addition, CNN is used throughout the algorithm to categorize the images that require closure operation and mask adjustment, providing a basis for choosing appropriate processing methods for different images, which improves the robustness and accuracy of the algorithm to a large extent.

### Experiment and evaluation

We divided the first set of images into training and validation sets in the ratio of 9:1. Training five CNN models on the training set, and all networks used SGD optimizer during training with an initial learning rate of 0.001 for 45 rounds. Finally, the five networks were tested on the validation set with accuracy and average precision (AP) as the evaluation criteria, and the network with the best classification results was used for the discrimination task of single- and multi-region distributions of the brain in FABEM.

We use the second set of images as the training set of five FCN models. The Adam optimizer is used uniformly during the training process. The initial learning rate is 0.001, and the training is 60 rounds. The 300 slices with the multi-regional distribution of the brain from the first set of images were selected as test set 1, and the third set of images was used as test set 2. The FCN model was tested on test set 1 after training. MPA (Mean Pixel Accuracy), MIoU (Mean Intersection over Union), and MBF (Mean boundary F1-Measure Score) [30] were used as evaluation metrics, where MBF uses 0.75% of the image diagonals as the tolerance distance. The model with the best segmentation effect was used for the segmentation of multi-regional distribution slices of the FABEM brain. Finally, five FCN models and FABEM were tested on test set 2. MPA, MIoU, MBF, and AET (Average Extraction Time) were used as evaluation metrics to further analyze various algorithms’ performance. Where AET denotes the average extraction time per slice in seconds/slice, and the test equation is:

4 $$AET=T/N$$

where, *T* denotes the total time to complete the extraction;

*N* denotes the total number of images;

The test set 2 is used as the test object and tested 5 times, and the results are averaged.

## Results

Table 1 demonstrates the classification results of each CNN network on head CT images. Among them, the accuracy and AP of the modified version of ResNet50 are the highest, reaching 99.6% and 99.74%, respectively; ResNet50 is the second highest, 0.4% and 0.76% lower than the modified version of ResNet50.The accuracy and AP of the modified version of AlexNet are 0.2% and 0.12% higher compared to AlexNet and VGG19, respectively. Therefore, the modified version of ResNet50 was used to discriminate head CT images in FABEM.

**Table 1:** Test results for each CNN algorithm

CNN Network	AlexNet	VGG19	Resnet50	Modified AlexNet	Modified Resnet50
Accuracy	0.9860	0.9860	0.9920	0.9960	0.9880
AP	0.9710	0.9710	0.9898	0.9974	0.9722

Table 2 shows the segmentation effects of the five models on test set 1. Among the three evaluation metrics, Deeplabv3+- ResNet50 (Deeplabv3 + model with ResNet50 as the backbone extraction network) has the highest MIOU and MBF values, which are 0.13% and 0.37% higher than the U-net model and 0.02% and 0.03% higher than Segnet Still, the MPA values were 0.01% lower than Segnet. It can be seen that Deeplabv3+- ResNet50 is better than the U-net and Segnet models for brain extraction of images with the multi-region distribution of the brain. Considering comprehensively, Deeplabv3+- ResNet50 was used to extract FABEM brain multi-region distribution images.

**Table 2:** Segmentation results of various FCN models in test set 1

Models	MPA	MIoU	MBF
U-net	0.9893	0.9825	0.9845
Segnet	0.9911	0.9836	0.9879
Deeplabv3+- ResNet50	0.9910	0.9838	0.9882
Deeplabv3+- Mobilenetv2	0.9903	0.9828	0.9873
Deeplabv3+- Xception	0.9904	0.9831	0.9872

Table 3 shows the test results of FABEM and the five FCN models in Test Set 2. Among the five FCN models, the MPA, MIOU, and MBF of Deeplabv3+- ResNet50 are the best, and the U-net is relatively low. Compared to Deeplabv3+- ResNet50, FABEM had 0.03% higher MPA, 0.15% higher MIOU, and 0.21% higher MBF. In addition, the AET of FABEM is much lower than that of the five FCN models, less than 26% of it.

**Table 3:** Test results of each algorithm in test set 2

Methods	MPA	MIoU	MBF	AET(seconds)
FABEM	0.9968	0.9936	0.9963	0.43
U-net	0.9957	0.9894	0.9825	1.69
Segnet	0.9963	0.9912	0.9929	1.74
Deeplabv3+- ResNet50	0.9965	0.9921	0.9942	1.68
Deeplabv3+- Mobilenetv2	0.9963	0.9914	0.9926	1.66
Deeplabv3+- Xception	0.9962	0.9918	0.9935	1.68

**Fig. 7:**
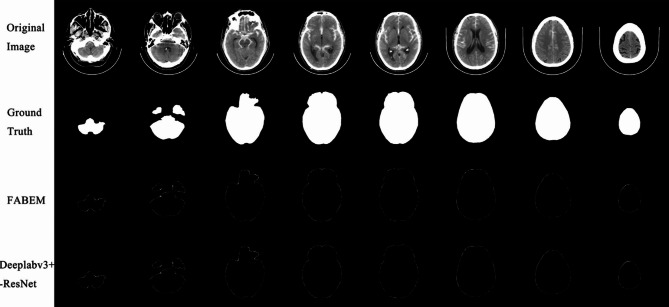
Absolute error between ground truth and the results of FABEM and Deeplabv3+- ResNet50

**Fig. 8:**
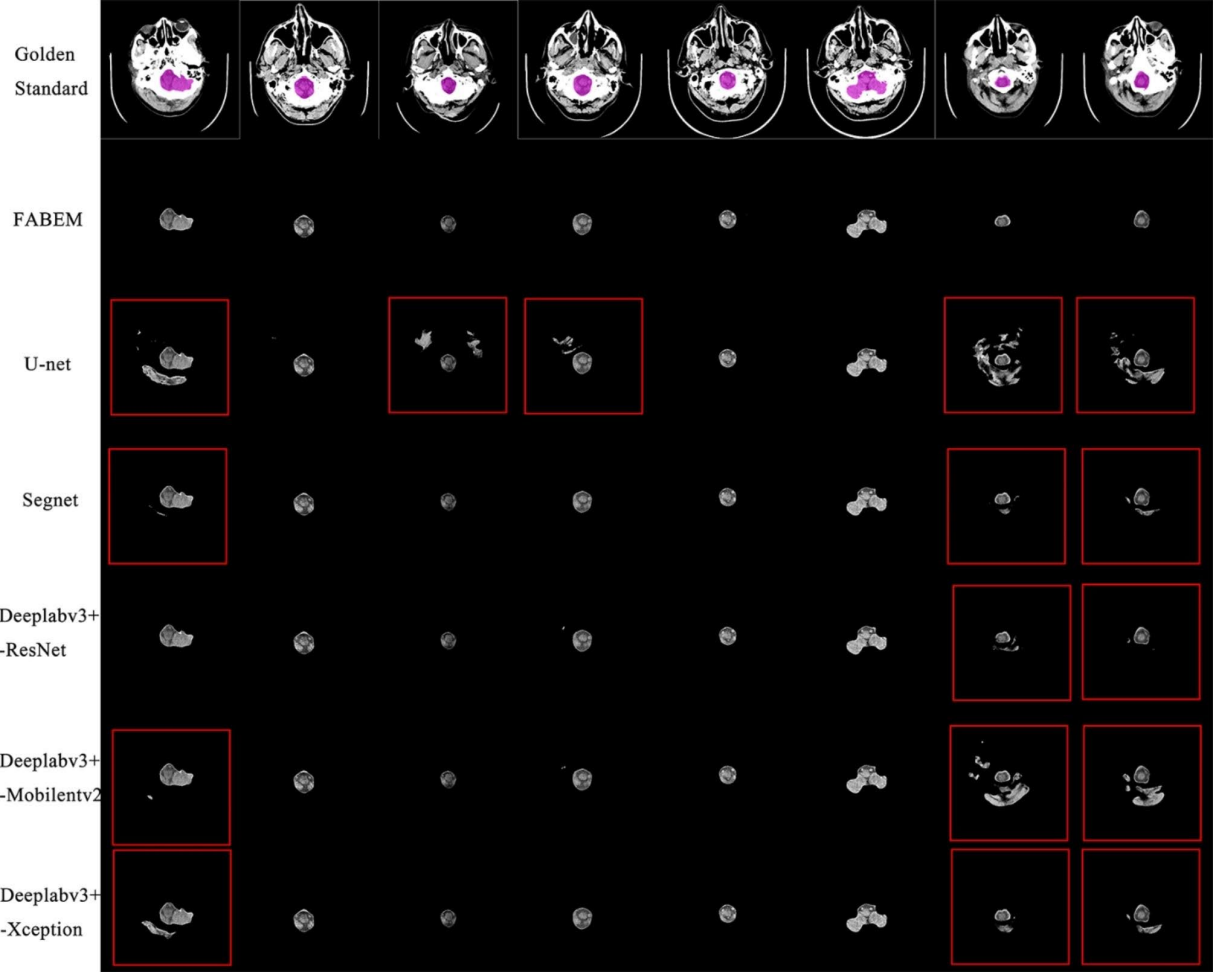
Extraction effects of each algorithm at the basis cranii layer

**Fig. 9:**
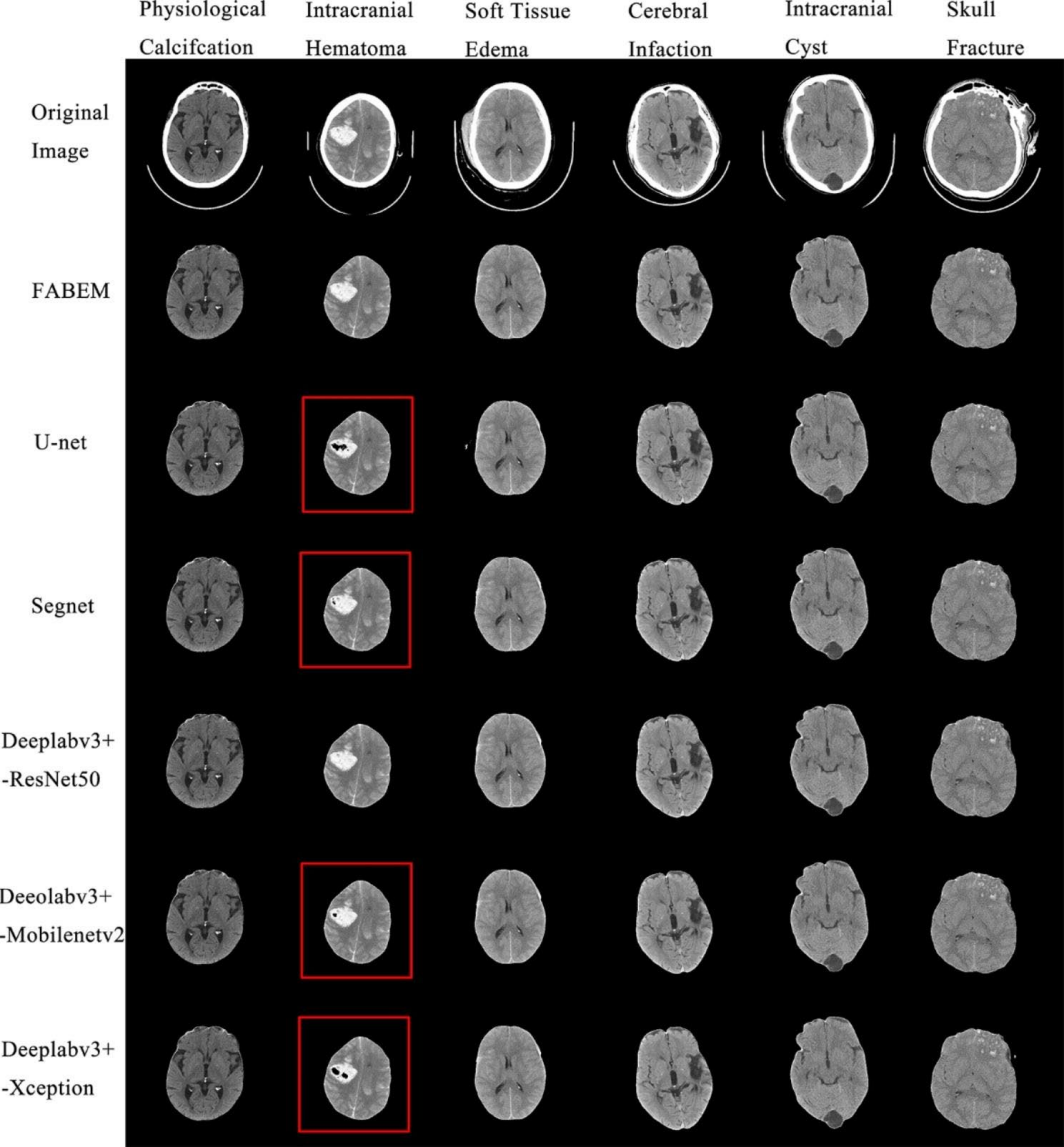
Segmentation effects of each algorithm on images containing different lesions

Figure 7 shows the absolute errors of FABEM and Deeplabv3+- ResNet50 concerning the ground truth of manual segmentation. There are eight images in total, which are selected from the cranial base to the cranial top in a group of head CT images. It can be seen from the figure that the error between the two and the ground truth is tiny. Figure 8 shows the extraction effect of FABEM and five FCN models in the basis cranii images. A total of eight images were randomly selected from test set 2. It can be observed that extracranial soft tissues are not removed to different degrees in all five FCN models. And no similar situation was found in the extraction effect map of FABEM. Figure 9 shows the effect of FABEM with the five FCN models on extracting images containing different lesions. It can be observed that physiological calcification, skull fracture, soft tissue edema, cerebral infarction, and intracranial cysts did not affect the five FCN models, but when faced with cerebral hematoma lesions, different degrees of missing lesion regions were found in the extraction effect maps of U-net, Segnet, Deeplabv3+- Mobilenetv2, and Deeplabv3+- Xception models. However, no similar situation was found in the extraction effect maps of FABEM and Deeplabv3+- ResNet50.

## Discussion

We present a fast and accurate brain extraction method for head CT images. Comprehensive analysis shows that FABEM can overcome the problems of soft tissue edema, unclosed skull, multi-region distribution of the brain, and complexity of basis cranii layer image structure compared with the existing traditional methods such as tracking algorithm, WMFCM, FCM, threshold segmentation, DRLSE, etc. [7–12] It possesses better robustness, ensures the accuracy of segmentation, and can be applied to the brain extraction of the whole set of head CT images. Compared with the secondary development of medical image post-processing software, FABEM is not dependent on specific software and is easier to integrate into other algorithms. Compared with FCN [27–29], this method achieves a segmentation accuracy close to FCN. Still, the segmentation speed is more than 3.8 times FCN, and it only takes about 0.43s to extract the brain from the head CT image using NVIDIA GeForce 3060Ti GPU, which improves the segmentation efficiency to a large extent. Comprehensive analysis shows that FABEM has better performance and is more in line with the current application requirements.

For the classification task of head CT images. Reducing the size of the original image and changing the input size of the convolutional neural network affects the classification accuracy for the head CT image classification task. Compared to the original network, the modified version of AlexNet shows a slight increase in accuracy and AP value. It also converges faster during training, which proves that the improved method is still effective, although limited. This is likely because the batch normalization layer enhances the network’s generalization ability and reduces the impact of changing the network input size. For the ResNet50 network model, changing the input size of ResNet50 produced better classification results than reducing the size of the original image, suggesting that changing the input size of the network produces relatively little negative impact.

For the task of brain extraction of multi-region distributed images of the brain, the segmentation speeds of the five FCN models are close to each other. However, the Deeplabv3 + model with ResNet50 as the backbone to extract the network has the best segmentation results, while the U-net model performs poorly compared to the other four FCN models.

For the effect of various lesions on the segmentation effect of each algorithm. According to the experimental results, FABEM and the five FCNs were not affected by the five lesions such as physiological calcification, skull fracture, soft tissue edema, cerebral infarction, and intracranial cysts, but U- net, Segnet, Deeplabv3+- Mobilenetv2, and Deeplabv3+- Mobilenetv2 showed a small area of absence in the region of cerebral hematoma. This may be because the brightness of the cerebral hematoma is close to that of the skull, resulting in the absence of this area. This is not the case with Deeplabv3+- ResNet50 because its backbone extraction network is more complex, with a larger number of parameters, which results in higher accuracy and robustness. FABEM, in its processing of the images, performs a second filling of the initial segmented image and chooses the Deeplabv3+- ResNet50 is chosen to accomplish the task of extracting images with the multi-region distribution of the brain, so this problem can also be well circumvented.

Considering comprehensively, we did not make obvious innovations in the network structure, but we designed special detection mechanisms, cycle structures, and an automatic seed point selection method to combine the algorithms of threshold segmentation, region growth, CNN, and FCN skillfully applied them to specific scenarios, and gave full play to the advantages of each algorithm [31, 32]. Especially for the task of brain extraction at the skull base level, our method outperforms FCN. in terms of segmentation quality and segmentation speed and achieves excellent results. In addition, we tested FABEM and five FCN models on datasets containing different lesions, explored each lesion’s influence on each algorithm’s segmentation effect, and drew relevant conclusions, which can be used as a reference for related research.

Our study also has some limitations. Since MR and CT systems are different imaging systems and their images are quite different [33], our study cannot be applied to the more sophisticated MR system. Of course, applying the method to MR will be our follow-up work.

## Conclusion

The method FABEM proposed in this paper combines the traditional image segmentation method with the FCN model, vastly improving the average extraction speed while ensuring good robustness and accuracy. It demonstrates the feasibility of combining the traditional image segmentation method with the FCN model to fully utilise both advantages in extracting the brain of head CT images. The corresponding contribution is providing an integrated algorithm to replace brain tissue’s manual segmentation. Moreover, the algorithm has a concise structure. It is easy to combine with related algorithms, which can provide a better environment for feature extraction of subsequent intracranial lesions and thus improve the speed and accuracy of following intracranial lesion recognition, location, and segmentation. However, this algorithm is not directly applicable to MR systems, and we will experiment and improve the algorithm for MR images in the later stage to expand its application scope.

### Electronic supplementary material

Below is the link to the electronic supplementary material.

Supplementary Material 1

## Data Availability

The datasets were derived from the RSNA Intracranial Hemorrhage Original Size PNGs (RIHOSP)dataset, publicly available on the Kaggle website(Login:a18737263685; Web Links: https://www.kaggle.com/datasets/vaillant/rsna-ich-png ) and the CQ500 dataset, publicly available on the Academic Torrents website(No registration number required; Web Links: https://academictorrents.com/details/47e9d8aab761e75fd0a81982fa62bddf3a173831 ). The datasets and materials used and analyzed in this study are available directly from the corresponding author.

## Abbreviations

FABEM: A fast and accurate brain extraction method
FCN: Full convolutional neural network
CNN: Convolutional neural network
CT: Computed tomography
MPA: Mean Pixel Accuracy
MIoU: Mean Intersection over Union
MBF: Mean boundary F1-Measure Score
AET: Average Extraction Time
