# Supplementary material for: A fast and accurate brain extraction method for CT head images
Source: BMC Med Imaging. 2023 Sep 12;23:124. doi: 10.1186/s12880-023-01097-0 (PMC10498619; doi:10.1186/s12880-023-01097-0)
Supplement: Supplementary file 1 — Supplementary Material 1 [file 12880_2023_1097_MOESM1_ESM.docx]

**Legend**

A total of two files have been uploaded for the supplementary material, both of which are described below:

Document 1：Source code for all algorithms_MATLAB code(.m)。

This file contains the source codes of all the algorithms, all of them are MATLAB code, which can be run directly on MATLAB2022b. It is divided into three parts; the first part is the source codes of CNN. There are 5 in total, which corresponds to the 5 CNN algorithms in the article, and the running of these programs needs a training set (CNN-traindata). The second part is the original code of FCN, 5 in total, corresponding to the 5 FCN algorithms in the article, and the training set (FCN-traindata) is required to operate these programs. The third part is the source code of FABEM. The operation of this program requires the support of two mathematical models, restnet2.mat and deeplabv3net50.mat.

Document 2：Source code for all algorithms_text file(.txt)

This document also contains the source code for all the algorithms in text format, with the same content as Document 1, and is intended to prevent access exceptions to Document 1.

The remaining supplementary material also includes the training set and two test sets for FCN, the training set, and test set for CNN, five trained FCN models, and five trained CNN models. However, these remaining files are too large, and most of the multiple attempts to upload them were unsuccessful. Readers can obtain these files by contacting the corresponding author directly. We are more than willing to share our datasets.
